# Cultural considerations: alcohol screening and brief intervention in a southern US level-1 trauma center

**DOI:** 10.1186/1940-0640-7-S1-A41

**Published:** 2012-10-09

**Authors:** Laura Veach, Regina Moro

**Affiliations:** 1 Department of Counseling, University of North Carolina at Charlotte, Charlotte, NC, USA

## 

This presentation addressed cultural considerations and sensitivities particularly relevant to the southern US when providing alcohol screening and brief intervention (SBI) to individuals demonstrating risky drinking behavior. Broader implications for education and training were also reviewed. The literature has begun to identify key issues pertaining to cultural sensitivities and considerations for successful change work with individuals. Key lessons learned about cultural considerations pertaining to alcohol SBI in one trauma center in the southern US were presented. Information included in the presentation was based on three years of alcohol SBI provided by counselors and doctoral and masters level student interns in a level-I medical trauma center. Implications regarding training SBI providers in cultural sensitivity were also discussed. To maximize an individual's acceptance of alcohol SBI, key cultural considerations may be implemented. In addition, educating and training SBI providers to enhance their cultural sensitivity is recommended.

